# Application and advances of biomimetic membrane materials in central nervous system disorders

**DOI:** 10.1186/s12951-024-02548-8

**Published:** 2024-05-23

**Authors:** Weiquan Liao, Zhichao Lu, Chenxing Wang, Xingjia Zhu, Yang Yang, Youlang Zhou, Peipei Gong

**Affiliations:** 1 Department of Neurosurgery, Affiliated Hospital of Nantong University, Medical School of Nantong University, Nantong, Jiangsu 226001 China; 2 Research Center of Clinical Medicine, Affiliated Hospital of Nantong University, Nantong, Jiangsu 226001 China; 3 Department of Trauma Center, Affiliated Hospital of Nantong University, Medical school of Nantong University, Nantong, Jiangsu 226001 China; 4 Jiangsu Medical Innovation Center, Neurological Disease Diagnosis and Treatment Center, Affiliated Hospital of Nantong University, Nantong, Jiangsu 226001 China

**Keywords:** Central nervous system diseases, Biomimetic membrane, Nanoparticles

## Abstract

Central nervous system (CNS) diseases encompass spinal cord injuries, brain tumors, neurodegenerative diseases, and ischemic strokes. Recently, there has been a growing global recognition of CNS disorders as a leading cause of disability and death in humans and the second most common cause of death worldwide. The global burdens and treatment challenges posed by CNS disorders are particularly significant in the context of a rapidly expanding global population and aging demographics. The blood-brain barrier (BBB) presents a challenge for effective drug delivery in CNS disorders, as conventional drugs often have limited penetration into the brain. Advances in biomimetic membrane nanomaterials technology have shown promise in enhancing drug delivery for various CNS disorders, leveraging properties such as natural biological surfaces, high biocompatibility and biosafety. This review discusses recent developments in biomimetic membrane materials, summarizes the types and preparation methods of these materials, analyzes their applications in treating CNS injuries, and provides insights into the future prospects and limitations of biomimetic membrane materials.

## Introduction

As medical technology advances, early interventions and treatment of CNS diseases have improved, thereby delaying disease onset and progression. However, despite these advancements, neurological diseases remain a primary cause of human disability and the second leading cause of death globally. Statistically, from 1990 to 2016, globally, the mortality rates of all neurological diseases and their disability-adjusted life-years (DALYs) combined have continued to increase, with a relatively large proportion of them, in order of prevalence, being strokes, migraines, Alzheimer’s disease and dementia, and meningitis, and a significant decrease in their age-standardized rates [1]. CNS disorders mainly include brain tumors, ischemic strokes, neurodegenerative diseases (Alzheimer’s disease, Parkinson’s disease, etc.) and epilepsy, presenting significant challenges to current medical technology. The growing incidence of CNS diseases not only compromises the health of many individuals but also imposes a substantial economic and social burden [2].

In addition, effective and comprehensive treatment strategies and therapeutic modalities for CNS disorders are currently lacking. Therefore, the innovation and design of effective and comprehensive therapeutic strategies and early diagnosis of CNS disorders are necessary. One of the major difficulties in the treatment of CNS diseases is to overcome the blood-brain barrier (BBB). Due to the BBB, most of the substances in the body’s circulatory system are blocked from entering the brain or enter the brain at very low concentrations, such as proteins, nucleic acids, drugs, body metabolites, fluorescent contrast agents and other macromolecules. Although the blood-brain barrier plays an important role in preventing damage to the brain parenchyma by blood-borne substances, it also seriously affects the early intervention and late treatment of CNS disorders with drugs and prognosis [3]. It is well known that non-lipid-soluble substances with molecular weights greater than 400 Da often fail to achieve pharmacologically significant drug concentrations due to the presence of the blood-brain barrier. Therefore, in order to improve the efficiency of the drug in crossing the blood-brain barrier, the constituent substances should be of low molecular weight, fat-soluble and easily dissociable.

There are three main biological barriers between the blood and the brain in the body: the BBB, the blood-cerebrospinal fluid barrier and the arachnoid barrier. The BBB consists mainly of microvascular endothelial cells in the capillaries with the largest area of material exchange between blood and brain. The cerebrospinal fluid barrier, composed of choroid plexus epithelial cells [4], can regulate the secretion of cerebrospinal fluid to the ventricular system. Among other things, cerebrospinal fluid is freely exchanged with interstitial fluid in several places. In contrast, the third barrier, which is on the arachnoid epithelium, because of its small surface area and lack of vascular distribution, mainly acts as a structural seal and has a minimal role in the exchange of substances between blood and brain [5].

To effectively treat these diseases, drugs need to cross the blood-brain barrier. To date, a number of nanoparticles (NPs) have been intensively investigated in laboratory studies and preclinical studies [6], such as organic and inorganic nanomaterials, polymeric NPs, carbon-based NPs, liposomes, and metallic NPs. However, there are some limitations of nanomaterials in the treatment of CNS disorders, such as biocompatibility, poor pharmacokinetics and cytotoxicity [7]. The specific manifestations are that NPs are prone to produce toxic polymers *in vivo;* abnormal adhesion and interaction with non-purpose cells, tissues or other cellular components [8]; abnormal metabolism in vivo, such as accumulation in the alveoli, brain, spleen, kidneys and other parts of the body, and even cause oxidative stress, which can produce cytotoxicity to the body and cause great damage to the organism. These abnormalities are usually closely related to the size, composition and route of administration of the NPs [9]. The above-mentioned disadvantages all seriously affect the passage of nanomaterials through BBB. Therefore, nanoparticle-based targeted drug delivery systems require further extensive research and development [10]. Interestingly, NPs wrapped in a biomimetic membrane(They are called biomimetic membrane materials) have received much attention for their natural biosurface, high biocompatibility, and biosafety [11], and can be used for the treatment of CNS diseases. Membrane-based NPs are increasingly being used because they can help drugs better penetrate the blood-brain barrier and enter the brain in a more targeted and efficient manner.

In recent years, biomimetic membrane materials have been developed and applied to CNS diseases, which can target the above limitations and shortcomings of nanomaterials. Biomimetic membrane materials are composed of different kinds of cell membranes wrapped around drug-containing nanomaterials, or extracellular vesicles (EVs, which are nanosized vesicles that can be secreted in almost all cells and have the same properties and functions as cell membranes, and which can be applied to the treatment of CNS diseases [12].) With the membrane biomimetic system, it is possible to cloak a drug in a “not eaten” form. Because the biomimetic membrane has high biocompatibility and biosafety, it can not only camouflage into the body’s intrinsic cells to avoid being captured by the immune system, thus prolonging the time in the blood circulation [13], but also has a variety of biological functions of cell membranes, such as low toxicity, targeting, penetration, etc., which may greatly improve the efficiency and concentration of the drug delivery into the brain.

In this review, the advantages and limitations of biomimetic membrane materials are introduced, the classification and preparation of biomimetic membrane materials are summarized, the characteristics of different central nervous system diseases are briefly introduced, as well as the therapeutic and application of biomimetic membrane materials in central nervous system diseases are summarized and analyzed. In addition, the future prospects and limitations of the biomimetic membrane material technology are also envisioned, which provides design methods and ideas for subsequent biomimetic membrane materials.

## Biomimetic membrane materials crosse the BBB

The way in which NPs (with small molecular weight) cross the blood-brain barrier (BBB) usually involves the following mechanisms [14]:

1. Active targeting mechanism: the transmembrane transport of nanomaterials is facilitated by modifying specific ligands or antibodies on the surface of the nanomaterials so that they can specifically bind to receptors on the BBB.
2. Passive diffusion: some nanomaterials may be able to diffuse passively through tight junctions between BBB cells or through non-specific pathways in the cell membrane due to their small size and specific physicochemical properties (like lipophilicity).
3. Endocytosis: Nanomaterials can be taken up by BBB cells through cellular endocytosis pathways (e.g., lattice protein-mediated endocytosis, vesicle protein-mediated endocytosis, microvesicle-mediated endocytosis, etc.), and then their loads can be released inside the cells.
4. Membrane fusion: certain nanomaterials are capable of fusing with the cell membrane, thereby releasing the load directly into the cell interior.

Since biomimetic membranes confer inherent cell membrane properties to NPs, their lipophilicity promotes the uptake of biomimetic membrane materials by endothelial cells on the BBB via a combination of caveolin-mediated endocytosis, clathrin-mediated endocytosis, micropinocytosis, and membrane fusion. In addition, ligands or antibodies on certain cell membranes and cellular homology enable specific binding, enhancing the targeting and crossing of the BBB by the biomimetic membrane materials [15, 16].

## Cell membrane-based nanomaterials

After an extensive literature review in recent years, it has been found that most of the cell membrane-based nanomaterials have been used in research for the treatment of CNS diseases, such as erythrocyte membranes, platelet membranes, neutrophil membranes, macrophage membranes, mesenchymal stem cell membranes, and cancer cell membranes, among others [17]. Recently, it has been found that more and more types of cell membranes are gradually being developed, such as the membranes of neural-type cells (astrocytes, microglia, oligodendrocytes, cortical neurons) and even brain endothelial cell membranes [16, 18]. They have been compared in terms of their efficiency in crossing the blood-brain barrier, and in addition to the pathway through the blood-brain barrier via the biomimetic membrane materials described above, their cellular homology greatly facilitates their easy passage through the blood-brain barrier. However, it is clear that the sources of these cell membranes are not easily accessible and are controversial in terms of practical clinical applications and moral ethics, as opposed to the commonly used cell membranes mentioned above. But in any case, these studies are a good discussion and reference for the therapeutic use of biomimetic membrane materials for drug delivery in central nervous system disorders.

### Cell membranes from different sources

#### Red blood cell membrane

The red blood cell membrane is a translucent structure composed of a bilayer of phospholipids and membrane proteins that have elasticity, stability and biocompatibility. The red blood cell membrane also expresses some immunosuppressive and self-recognizing proteins, such as CD47, which can inhibit phagocytosis by macrophages and prolong the circulation time of red blood cells in the blood [19].

Red blood cell membranes can be fused with polymer NPs by mechanical extrusion to form biomimetic nanomaterials with a core-shell structure. Such NPs can mimic the long-circulating properties and functions of red blood cells for drug carriers [20]. The bilayer of phospholipids in the red blood cell membrane can provide a stable shell that prevents excessive drug release and serum aggregation. Membrane proteins on the red blood cell membrane can maintain their original structure and activity, providing immune escape and targeted delivery capabilities for NPs. The source of red blood cell membranes also allows for personalized drug delivery avoid immune rejection and allergic reactions.

#### Platelet membrane

Platelets are nucleated blood cells that are primarily responsible for hemostasis and thrombosis. The membrane of platelets is composed of a phospholipid bilayer, proteins, and sugars that express a variety of receptors and molecules involved in platelet activation, adhesion, and signaling. Platelets play an important role in normal physiological functions as well as in a range of pathological processes such as cancer, inflammation and atherosclerosis. Platelet membranes can be used to encapsulate NPs to form a biomimetic drug delivery system [21]. Such systems are highly biocompatible, immune escape and targetable and can effectively deliver drugs to damaged blood vessels, tumor cells and inflammation sites. Platelet membranes can also be used to prepare hybrid membranes that combine the functions of cells of different origins to enhance the multifunctionality of NPs. NPs coated with platelet membranes have shown promising applications in cancer therapy [22], immune disease treatment [23], atherosclerosis treatment [24]and phototherapy [25].

#### Neutrophil membrane

Neutrophils are derived from myeloid progenitor cells commonly found in bone marrow and extramedullary tissues (e.g., spleen), and account for 50–70% of the total leukocytes in the peripheral blood of humans (only 10–25% in mice) [26]. Neutrophils are important immune cells that can migrate across the blood-brain barrier by chemotaxis to reach inflamed brain areas and participate in inflammatory responses and tissue repair. With specific receptors and ligands on their surface, they can interact with endothelial cells of the blood-brain barrier for transmembrane transport [27]. However NPs encapsulated by neutrophil membranes also have limitations as it has been found that activated neutrophils can lead to secondary inflammatory injury because neutrophils release reactive oxygen species (ROS), bioactive lipid mediators, and neutrophil extracellular traps (NETs) at the site of inflammation [28]. Therefore, this should be taken into account when selecting this cell membrane for making biomimetic membrane materials.

#### Macrophage membrane

Macrophages are important leukocytes involved in non-specific and specific immunoregulation, belonging to the class of monocytes, which remove foreign substances and waste products from the organism mainly by phagocytosis of bacteria, dead cells or cell debris. Macrophages can adapt to changing environments by changing their morphology and physiological functions, which is known as macrophage polarization [29]. The cell membranes of polarized macrophages with different phenotypes have unique biological functions that greatly expand the biological applications of macrophage membranes. Macrophages are important immune cells involved in inflammatory responses and cell recruitment, and their membranes express a variety of functional proteins, such as CD47 and integrin α4/β1. CD47 can bind to SIRP-α receptor and inhibit macrophage phagocytosis by the monocyte system (MPS). And integrin α4/β1 can specifically bind to vascular cell adhesion protein 1 (VCAM-1), which in turn promotes macrophage aggregation in atherosclerotic plaques [30]. It has been shown that macrophage membranes can likewise play a good role in the treatment of spinal cord injuries by improving the efficiency of drug delivery [31].

#### Mesenchymal stem cell membrane

Mesenchymal stem cells (MSCs) are a class of pluripotent stem cells found mainly in connective tissues and organ mesenchyme, including bone marrow, umbilical cord, adipose, mucous membranes, bones, muscle, lung, liver, pancreas and other tissues, as well as amniotic fluid, amniotic membrane, and placenta, etc [32]. Under appropriate conditions, MSCs have strong multidirectional differentiation potentialand can differentiate into various tissue cells such as bone, cartilage, fat and so on. In addition, MSCs have the ability to self-replicate and can promote hematopoiesis and immunomodulation [33]. Therefore, MSCs are considered to be the closest stem cell product to clinical application. In addition, MSCs are tumor-tropic, and due to this homing property, they have strong utility in designing of drug carriers for tumor-targeted therapies [34]. It has been shown that several pairs of chemokine ligands and their receptors are involved in this chemotaxis. For example, SDF-1 and its receptor CXCR4 (SDF-1/CXCR4), platelet-derived growth factor and its receptor (PDGF/PDGFR), vascular endothelial growth factor and its receptor (VEGF/VEGFR), and so on [35]. Tumor cells can secrete these specific chemokines that bind to the corresponding receptors on MSCs. Therefore, wrapping MSC membranes around drug-containing NPs can be used to achieve good targeting by exploiting their natural homing properties, which in turn increases the drug concentration at the target site. Interestingly, the expression of MHC class II molecules is very low on MSCs, which makes MSCs targeting of tumors not species-specific [36]. Therefore, MSCs from different species origins may also be available for clinical treatments.

#### Cancer cell membrane

Cancer cell membrane is a membrane on the surface of cancer cells, consisting of a phospholipid bilayer and membrane proteins. Cancer cell membrane camouflages NPs, by coating cancer cell membrane, NPs can mimic the surface characteristics of cancer cells, thus avoiding recognition and removal by the immune system and prolonging blood circulation. In addition, cancer cell membranes can also enhance targeting ability. Through the cancer cell membranes, NPs can obtain cancer cell-specific membrane antigens, such as N-calmodulin, galactoglucan-3, or epithelial cell adhesion molecules [37], which have the ability of homotypic binding and can bind specifically to homologous cancer cells, to achieve precise tumor-targeted delivery [38, 39]. Through the cancer cell membrane, NPs can retain some biological functions of cancer cells, such as extravasation, chemotaxis, and cancer cell adhesion, which can help NPs to penetrate the tumor microenvironment and increase the penetration and effectiveness of drugs. Therefore, tumor cell membranes can provide a good idea for nanoparticle design in the field of tumor diagnosis and therapy [40, 41].

### Design and preparation of cell membrane-based nanomaterials

In this context, biomimetic membrane materials made of various nanomaterials combined with biomimetic membrane can overcome the shortcomings of traditional nanomaterials to a large extent [42]. Currently, the fabrication strategy for cell membrane-based nanomaterials is mainly to utilize cell membranes to encapsulate nanoparticles [43]. By camouflaging cell membranes, drug-loaded NPs can possess the inherent properties of external cell membranes, such as invisibility to the immune system, penetration of the blood-brain barrier, and so on. Cell membrane-based nanomaterials with these biological properties are not only more biocompatible, but also have better diagnostic and therapeutic efficacy in vivo.

Considering the successful examples that have been prepared previously [21], the preparation of cell membrane-based NPs is very similar and is divided into three main steps: (1) extraction of cell membranes; (2) preparation of NPs; and (3) fusion of cell membranes with NPs.

#### Cell membranes extraction

The extraction of cell membranes is subdivided into the extraction of anucleate cell membranes and the extraction of nucleated cell membranes.

Red blood cells are a typical example of anucleate cells, so the red blood cell membrane was also one of the first cell membranes to be used for coating NPs. First, fresh whole blood was obtained from the animals and centrifuged at 4 °C to remove the serum and the leukocyte layer, and the red blood cells were collected; the red blood cells were washed repeatedly with phosphate buffered saline (PBS) and centrifuged again to remove residual plasma and other unwanted cells; Red cell membranes were prepared by hypotonic treatment, that is, washed red cells were gently mixed with an excess of 0.25× PBS, left to stand, and then released from the red cells; hemoglobin was removed by high-speed centrifugation, and the red cells were collected in the pink precipitate; followed by ultrasonication with a bath sonicator, and then red cells were extruded with different pore sizes of polycarbonate porous membranes through the Avanti microextruder, to obtain the target size of erythrocyte membrane vesicles. Finally, protease inhibitors were added to maintain the bioactivity of the membranes and stored at 4 °C for cryopreservation [20, 44].

The extraction of nucleated cell membranes becomes more difficult due to the complexity of the nucleated cell interior, which has been summarized in a large number of literature, and the methods and steps for the extraction of nucleated cell membranes are very similar, and the main method used is cell lysis by hypotonic treatment combined with repeated freeze-thaw cycles [45, 46]. Differential centrifugation was then used to remove the complex components from the cells, and it was found that better results were also achieved using discontinuous sucrose gradient centrifugation [47] and membranes was obtained. Lastly, a specific buffer is applied to purify the resulting cell membrane [38].

In addition, there are many other methods for lysis and extraction of cell membranes, such as mechanical techniques: high-pressure homogenizer method, microbead milling method, etc.; and non-mechanical techniques: three main categories: physical, chemical and biological. (1) Physical destruction is a non-contact method of destroying cell membranes using external forces. For example, cell membranes are cleaved using heat, sound and pressure. (2) Chemical lysis involves the use of a lysis buffer to disrupt the cell membrane. Lysis buffers disrupt cell membranes by altering the pH. Detergents can also be added to the cell lysis buffer to dissolve membrane proteins and rupture the cell membrane to release its contents. (3) Biologically lysed cells are mainly cleaved by various biological enzymes, such as lysozyme, staphylococcal lysozyme, protease, etc. Each method has its advantages and disadvantages, so the most appropriate lysis method must be selected based on the type of cell membrane extracted and the purpose [48].

#### Preparation of NPs

NPs were prepared as follows:

1. Organic NPs (poly (ethylene imine), poly (ethylcyanoacrylate), poly (lactide-co-glycolic) acid, etc.) and inorganic NPs (gold nanoparticles, silica nanoparticles, carbon nanotubes, etc.) are prepared by different synthetic methods, such as chemical reduction, sol-gel, hydrothermal, and microemulsion methods [49].

(2) Nanoemulsions are prepared by mixing two immiscible liquids into a single phase, which requires the use of an emulsifier to stabilize the emulsion [50].

(3) Nanocrystals are prepared by modifying the surface of solid carriers (usually spherical) that are usually negatively charged, amorphous and hydrophobic [51].

(4) Gold quantum clusters are prepared by aggregating gold atoms into small clusters that have quantum-limited domain effects and fluorescent properties.

The morphological type and size of NPs have a strong influence on cellular uptake and excretion [52]. We can also deliver NPs to the target site by changing the mode of administration of NPs. (such as oral, subcutaneous, transdermal, and nasal inhalation, etc.)

#### Fusion of cell membranes with NPs

Fusion process: after mixing the cell membrane vesicles and the inner core nanocarrier, the cell membrane is wrapped around the surface of the inner core nanocarrier by extrusion, ultrasonication, or electroporation [53] to form biocompatible and biofunctional cell membrane-coated NPs [54]. Among them, membrane extrusion and sonication bath are two of the most commonly used methods [55]. For cell membrane extrusion, both membrane carriers and inner core nanocarriers can be repeatedly extruded several times through nanoscale polycarbonate porous membranes using an Avanti miniextruder. In this extrusion process, the cell membrane is wrapped around the NPs by mechanical force. This method is simple and convenient, but difficult to produce in large quantities. The sonication bath is a method capable of high throughput, which precisely solves the above problems and is suitable for large-scale production. However, the technical requirements of the sonication bath are much higher than those of membrane extrusion, and improper control of the power of the sonication process and excessively high temperatures may affect the destruction of the structure of the surface proteins of the obtained cell membranes, thus affecting the biological function of the cell membranes of the biomimetic membrane materials after fusion; in addition, the size of the membrane vesicles produced by the sonication bath is not homogeneous [54]. Therefore, most laboratories choose the former route for the preparation of biomimetic membrane materials.

## Extracellular vesicles(EVs)

### Biogenesis of extracellular vesicles

Extracellular vesicles can be categorized as exosomes, microvesicles, and apoptotic vesicles, differentiated according to their size, origin, and composition [56].

Biogenesis of exosomes: Exosomes are the smallest EVs, with a size of about 100 nm, and their generation is divided into three stages: plasma membrane invagination to form endocytosed vesicles, some endocytosed vesicles to form early sorting endosomes (ESE), exchange of substances between ESE and cell contents or fusion of ESE and cellular contents to form late sorting endosomes (LSE), and LSE eventually evolve into multivesicular bodies (MVB), and then fuse with the cell membrane to release small vesicles, which are exosomes. Many important proteins are involved in this biogenesis progress and play important roles in it, such as endosomal sorting complex required for transport (ESCRT) proteins, transmembrane proteins (CD9, CD63, CD81), heat shock proteins, RAB GTPase proteins, and SNARE protein complexes.

Biogenesis of microvesicle: Microvesicles are produced through outward budding and fission of the plasma membrane and are 20–1000 nm in size. The asymmetric distribution of phospholipids in the bilayer of the cell membrane, calcium efflux, phospholipid scramblase, and the ARRDC1 protein plays an important role in the biogenesis process of microvesicles.

Biogenesis of apoptotic vesicles: Apoptotic vesicles are large vesicles formed by apoptotic cells, with a size of 50–5000 nm, containing cytoplasm, organelles, and nuclear fragments, which are the result of blistering of the plasma membrane in the process of apoptosis.

### EVs as a drug carrier in the central nervous system

EVs are nanoscale membrane vesicles that can be secreted by almost all cells and can be categorized into three types: exosomes, microvesicles, and apoptotic vesicles. The membranes of EVs can carry a variety of biomolecules, including membrane proteins, miRNAs, and so on. EVs serve as important mediators for cell-to-cell communication, and contribute to their development and function, and play important roles in physiological and pathological processes. As “molecular carriers”, EVs can be used as novel tools for various therapeutic and diagnostic purposes, such as antitumor therapy, immunomodulation and drug delivery. Recently, EVs have been found to be involved in the pathogenesis of neurodegenerative diseases [56], and can be used as potential therapeutic targets or biomarkers for neurodegenerative disorders [57]. Compared with traditional drug delivery vehicles, EVs have great potential and advantages as drug carriers due to their high biocompatibility and low immunogenicity. In addition, EVs can act as transfer carriers for membrane receptors and fulfill the functional roles of their contents such as growth factors and nucleic acids [58]. Using this specific delivery, we can load some small molecules (drugs, viruses, NPs, etc.) into EVs and transport them to specific sites. Thus, we can improve the disadvantages of traditional drugs such as low concentration at the site of injury, premature release during transportation, phagocytosis by the immune system, and so on. For example, it has been reported that brain endothelial cell-derived EVs loaded with the anticancer drugs paclitaxel and adriamycin can reach the tumor site for therapeutic purposes via the BBB in a zebrafish model [59]. In addition, pituitary adenylate cyclase-activating polypeptide (PACAP) and estrogen can be encapsulated in nanogels and used for the treatment of perimenopausal depression via EVs carriers [60]. There are also reports indicating that M2-type primary peritoneal macrophage-derived EVs can be used as a drug carrier for berberine (Ber) in a mouse model of spinal cord injury [61].

Although almost all types of cells can secrete EVs, there are strict criteria and requirements for the selection of drug carriers, such as carrier yield, space size, surface proteins and internal components. To date, a variety of cell types have been selected as donors for EVs and have been used in experimental studies for the treatment of CNS disorders, including CNS cells [62], erythrocytes, macrophages, tumor cells, dendritic cells, mesenchymal stem cells, and immune cells, among others, which have a great potential for drug carrier applications [63]. It has been found that unmodified EVs of different cellular origins were injected into the vein, and it was observed that extremely few EVs were delivered to the brain of rats [64, 65], which is likely that EVs inherited the properties of the parent cells from which they originated and selectively avoided the blood-brain barrier to reach the brain. Recent studies have shown that EVs secreted by different cells are highly variable in their tropism for each organ or tissue, and therefore, this feature can be exploited to selectively screen EVs of different origins, so that EVs can be used as drug carriers in the central nervous system. For example, in a stroke mouse model, comparing the EVs of neural stem cells to those of MSCs, the former secreted EVs that significantly enhanced targeted delivery to the brain [66]. It has also been found that in mouse models of cerebral hemorrhage or traumatic brain injury (TBI), miR-143-3p carried in astrocyte-derived EVs was found to promote peripheral neutrophil migration across the BBB to the site of injury, and this phenomenon was exploited to select astrocyte-derived EVs to carry drugs to inhibit this pathway for therapeutic effect. The tropism of EVs differences are also likely to be influenced by the physiologic or pathologic environment *in vivo.* For example, in a mouse model of brain inflammation, the accumulation of macrophage-derived EVs in the brain after 10 min of brain inflammation was 5.8 times higher than that of healthy mice [67]. This may be because EVs inherit LFA-1 of macrophages, which is a protein that interacts with ICAM-1 of endothelial cells and mediates the lateral migration and dialysis of macrophages across the BBB [68], thus enhancing the interaction between EVs and brain endothelial cells and crossing the BBB. It has also been shown that in an in vitro stroke model, EVs derived from HEK293T cells activated with TNF-α are more easily internalized by brain endothelial cells and enhance EVs penetration in the BBB [69].

However, not all EVs are suitable for drug carriers. In the pathological setting, many EVs are playing the role of villains, exacerbating the hostile environment of the disease. For example, in a mouse model of TBI, EVs are significantly increased in the peripheral blood, and highly express pro-coagulant TFs on their surface and carry various inflammatory mediators, such as inflammatory vesicles, to amplify the inflammatory response, leading to lung epithelial cell death and blood-gas barrier disruption, and even affecting coagulation [70], In addition, brain-derived EVs activate leukocytes and platelets in the induction of systemic coagulation disorders after TBI and inflammation [71, 72]; in epileptic mechanisms, it was found that epileptogenic neuron-derived EVs carrying miR-181c-5p reduced glutamate uptake capacity of astrocytes and promoted susceptibility to epilepsy [73]. Therefore, before selecting EVs as drug carriers, the source of EVs and the physiological and pathological environment of the animal model must be considered; EVs from different sources have different biological functions and targeting sites, which will affect the efficiency of drug delivery.

### Design and preparation of Evs

#### Isolation of EVs

Ultrafast centrifugation is a classical method for EVs separation and one of the reported and most commonly used extraction techniques [74]. Ultracentrifugation is a kind of physical separation method, which has little effect on the biochemical structure and physiological function of EVs and is relatively simple to operate. However, EVs may also be damaged by excessive rotational speed and affect the activity of its contents (such as DNA, RNA, etc.). The general process is as follows: First, expand the culture of the mother cells, wait until the growth of the mother cells to about 85%, then discard the medium, wash the cells with sterile PBS three times, and then add the serum-free medium to culture the cells for 48 h, will get the cell culture supernatant containing the EVs secreted by the mother cells. Then the collected supernatant was centrifuged at 500 g at 4℃ for 5 min and the dead cells were removed. Then centrifuge at 2000 g and 10,000 g for 30 min and 60 min respectively at 4℃ to remove cell debris; Finally, EVs was obtained after 120,000 g centrifugation at 4℃ for 1 h. In order to purify EVs, EVs can be re-suspended with sterile PBS, filtered with 0.22µM sterile filter, re-centrifuged at 120,000 g at 4℃ for 1 h, and then re-suspended in sterile PBS or RIPA buffer and stored at -80 °C until further use. In addition to ultracentrifugation, other methods for extracting EVs include ultrafiltration [75, 76], size exclusion [77], polymer-based precipitation [78], immunoaffinity capture [79] and microfluidics [80]. Ultrafiltration is a technique that utilizes the difference in pressure across the ultrafiltration membrane and separates EVs based on their characteristic size; size exclusion is a technique that allows the sample to flow through the column, where substances larger than the pore size of the gel particles are not allowed to enter the pores, and they elute through the space between the porous gel and the mobile phase; polymer co-precipitation technique is based on the principle that hydrophilic polymers interact with the hydrophilic bonds of the sample’s EVs to form a hydrophobic microenvironment around the EVs, which results in the formation of precipitates and extraction of EVs; the immunoaffinity capture technique is based on the principle of isolating and enriching EVs by identifying specific proteins of EVs; and microfluidics is a signal-detection-based technique for EVs extraction. Although there are very many ways to extract EVs, it is necessary to consider the method of extracting EVs for your own experimental requirements due to the low purity of the extraction, overly complex operation, disruption of the sample structure, high cost and low yield.

#### Loading of EVs

**Scheme. 1:**
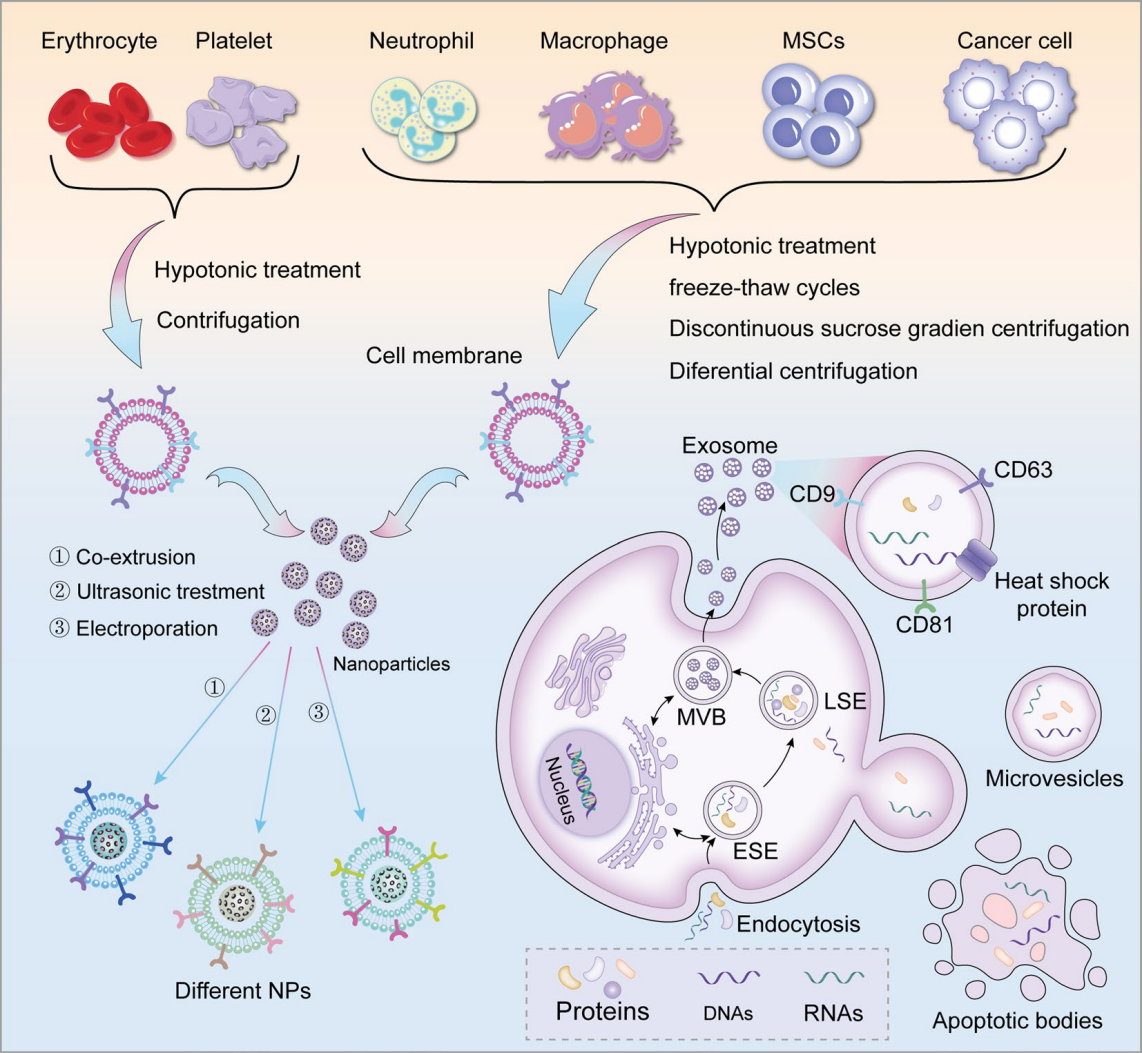
The Schematic diagram of the synthesis process of biomimetic membrane materials

According to the studies reported so far, the loading of EVs is divided into two main categories, one of which is the use of electroporation or ultrasound to make EVs loaded with drugs or other small molecules (e.g., microRNAs [81], proteins [82], etc.), but this physical method will cause some rupture to the EVs membrane. Thus, some studies have shown that it is possible to load EVs with small molecules by natural uptake pathways. For example, an active drug, paclitaxel, was used to co-culture with MSCs, and its derived EVs could carry the drug to inhibit tumor cell growth in vitro [83]. By this loading method, EVs can be easily recovered because EVs containing carriers can be easily precipitated by ultracentrifugation, which can better maintain the natural properties of EVs surface proteins [84]. However, the exogenous methods of electroporation or sonication are still more commonly used in laboratory studies (Scheme 1).

## Application of biomimetic membrane materials in different CNS diseases

The majority of CNS disorders are incompletely curable, including various types of spinal cord injury (SCI), stroke, Alzheimer’s disease (AD) and CNS tumors. To date, a variety of biomimetic membrane materials have been used as ideal tools for the treatment of CNS diseases.

### Spinal cord injury

Spinal cord injury is a neurological condition usually caused by violent shock. Injuries caused by violent shock acting directly on the spinal cord are called primary injuries, whereas on the basis of primary injuries, oxidative stress or apoptosis occurring around the site of injury leading to inflammatory injuries are called secondary injuries. These processes can expand the damaged area of neural tissue and exacerbate the loss of neurological function, ultimately leading to paralysis. The global incidence of spinal cord injury has been reported to be 10.4–83 cases per million/year, and it usually leads to severe long-term disability as it causes many patients to lose the use of their arms, legs, bowels, bladder and sex, among others. This injury is more common in males with a high incidence peak in the age of 30 years [85], but there is an increasing proportion of acute spinal cord injuries in the elderly as a result of falls. Due to the existence of the blood-spine-brain barrier, drug delivery for spinal cord injuries often fails to achieve the therapeutic effects at the desired drug concentration, and the emergence of biomimetic membrane materials provides new therapeutic strategies for spinal cord injuries.

**Fig. 1:**
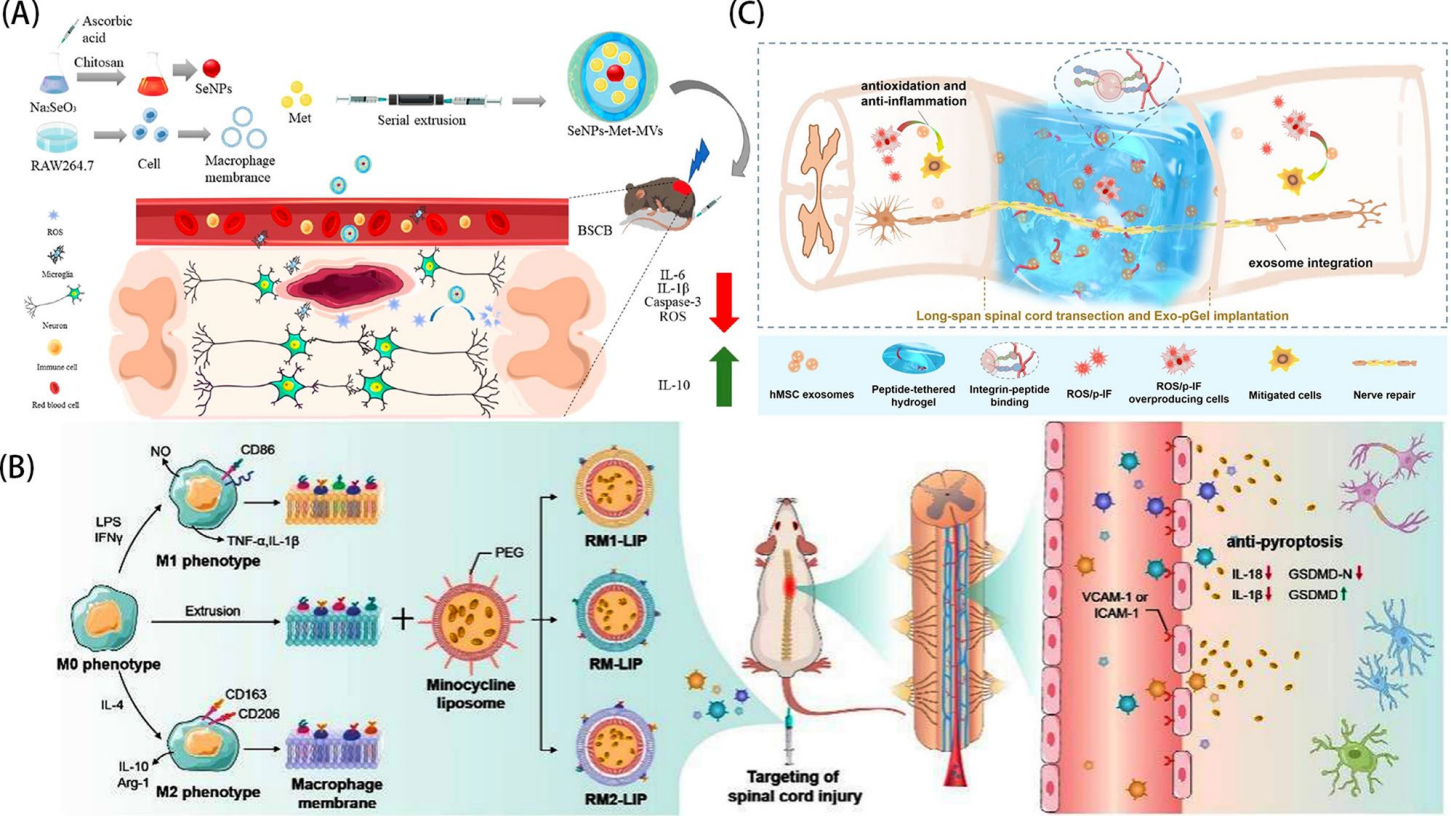
(**A**) The preparation of SeNPs-Met-MVs and their mechanism of action in the treatment of spinal cord injury. (**B**) The preparation of RM-LIP and its action mechanism for targeted drug delivery in the treatment of spinal cord injury. (**C**) Schematic diagram of Exo-pGel for the treatment of spinal cord injury. Reprinted with permission from Liu et al. [86], Tang et al. [31], Li et al. [90]

Liu et al. [86] developed a biomimetic membrane material (SeNPs-Met-MVs), in which selenium nanoparticles (SeNPs) and metformin (Met) are encapsulated with nanovesicles secreted by macrophage membranes (MVs)(Fig. 1A) As integrin lymphocyte function-associated antigen 1 (LFA-1) on macrophage membranes can bind to cell adhesion molecule-1 (ICAM-1) in endothelial cells [87], which promote the targeting and passage of SeNPs-Met-MVs through the BBB, and because of the properties of macrophage membranes, it is easy to be convened to the site of inflammation, furthermore, because of the macrophage membrane’s biocompatibility and biologically safety, SeNPs-Met-MVs evaded capture by the immune system, prolonged the action time of the drug Met at the site of inflammation, and successfully crossed the blood-spinal cord barrier to reach the site of spinal cord injury. These properties ensure that SeNPs-Met-MVs can successfully cross the blood-spinal cord barrier and target to the inflammatory site of spinal cord injury and exert therapeutic effects, so macrophage membranes are well suited to serve as biomimetic membrane carriers for pharmacological treatments of spinal cord injury [31].

Tang et al. [31] also used macrophage membranes mixed with polyethylene glycol-modified liposomes (PEG-LIP) containing minocycline and prepared RM-LIP by extrusion (Fig. 1B). They not only explored the therapeutic effect of RM-LIP on spinal cord injury, It was also considered that macrophage membrane might express different target receptors under different polarization conditions, which might affect the efficacy of RM-LIP delivery. By comparing the expression of integrin α4 and Mac-1 on the membrane of the three subtypes of macrophages, it was found that the different polarization states had no significant effect on the expression of the main target receptors on the membrane of macrophages and were effective in treating SCI mice. This research strategy provides a new idea for considering the effect of cell typing on NPs before the design of biomimetic membrane materials, and also validates the great practicability of macrophage membrane for drug delivery in spinal cord injury. At the same time, we can also take advantage of the characteristics of macrophages being easily recruited to the inflammatory site, and consider the use in other central nervous system diseases that resemble the microenvironment, such as traumatic brain injury (TBI), meningitis, etc.

In addition, the transplantation of MSCs has been applied to the treatment of spinal cord injury in reported studies [88], and its mechanism of action is attributed to the paracrine role of MSCs, whose derived EVs can carry various biomolecules of their cells of origin including membrane proteins, miRNAs, and so on, which are important mediators of intercellular communication, and EVs happen to play an important role here [89].Li et al. [90] provided new ideas for the use of EVs in the treatment of spinal cord injuries. They used a hydrogel containing hydroxy-acetylated hyaluronic acid (HA) as a carrier for exosomes derived from human mesenchymal stem cells and introduced an adhesive peptide PPFLMLLKGSTR derived from laminin through modification to enhance the affinity between the hydrogel and exosomes. By enabling the effective adsorption of exsomes into the porous structure of the hydrogel, an exosome hydrogel complex (Exo-pGel) was formed, (Fig. 1C) and the Exo-pGel was implanted into both ends of the severed spinal cord in the spinal cord amputation model to form a bridge similar to the extracellular matrix, which provided physical support and biostimulation for spinal cord repair. Therefore, based on such a drug delivery strategy, in spinal cord injuries or other CNS diseases, we can draw on stem cell transplantation protocols and use the corresponding cell-derived EVs to design new drug delivery protocols.

### Ischemic stroke

Ischemic stroke is a series of vascular diseases that lead to brain necrosis due to insufficient blood and oxygen supply to brain tissues. Its main cause is the narrowing or occlusion of cerebral arteries due to cerebral atherosclerosis, while other causes include congenital vascular malformations, cerebral embolisms, infections, and blood disorders [91]. Ischemic stroke affects mainly middle-aged and elderly people and is one of the most common types of cerebrovascular disease (about 70% of all acute cerebrovascular diseases). The symptoms of ischemic stroke vary according to the site of infarction, size, and degree of vascular obstruction [92], and can lead to severe consequences such as paralysis, aphasia, and blindness. Cerebral infarction is currently one of the leading causes of death in China. Due to the presence of the BBB, it is difficult for conventional medications to be transported to the site of injury, which makes the treatment of ischemic stroke more challenge. Therefore, there is a need to search for drugs or drug carriers that can effectively cross the BBB and increase distribution in the brain. Nanodelivery systems can cross the BBB by passive diffusion, endocytosis, receptor-mediated active transport, drug carrier transport, etc. Nanocarriers for brain-targeted delivery can also be constructed using natural polymers, synthetic polymers, inorganic materials with extracellular vesicles, and other biomimetic membrane materials. Nanodelivery systems provide new ideas and prospects for the treatment of ischemic stroke [93].

**Fig. 2:**
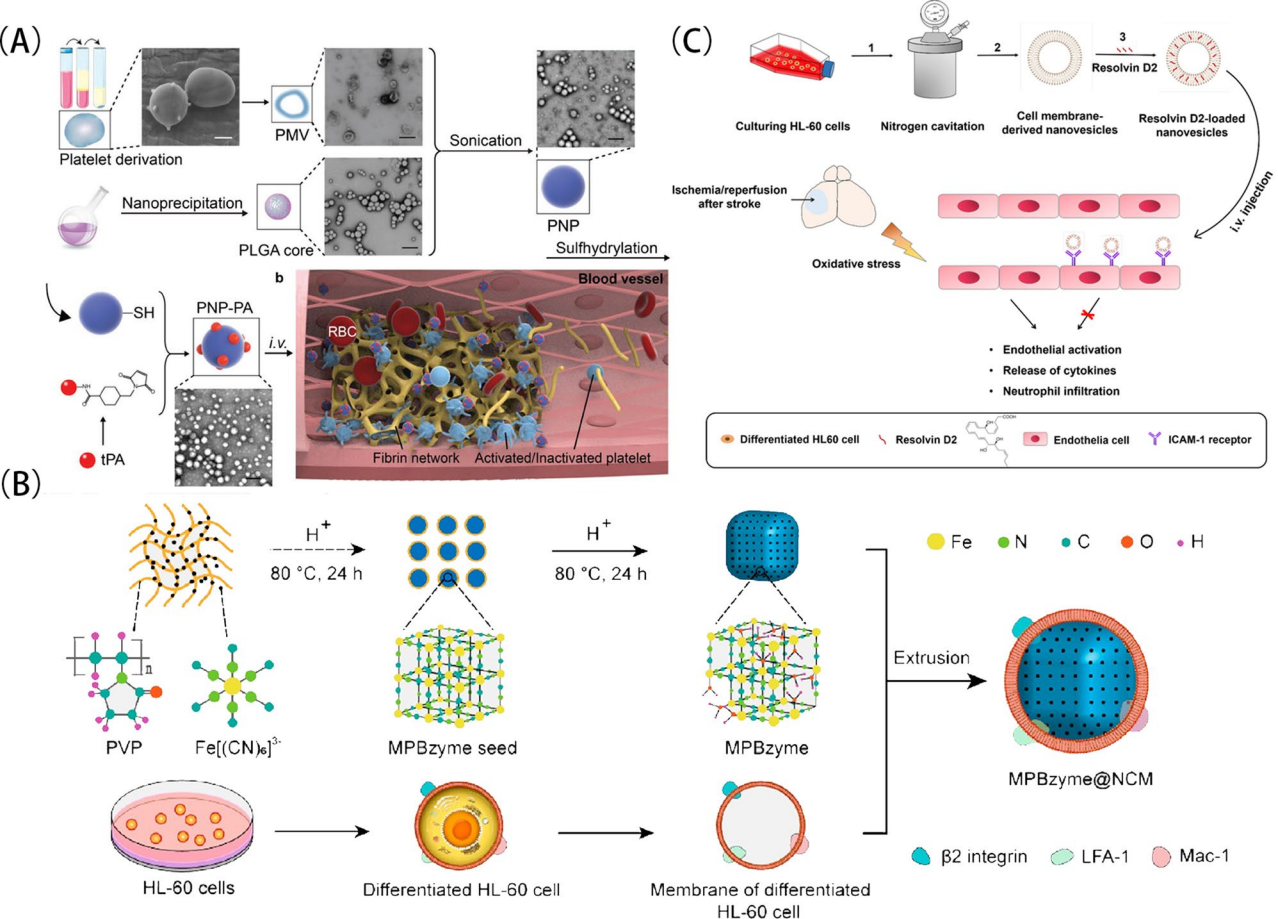
(**A**) The schematic diagram of synthesis of PNP-PA nanoparticles and its mechanism of action in thrombolysis. (**B**) The illustration of the synthesis of MPBzyme@NCM and its treatment of ischemic stroke. (**C**) The preparation process of RvD2-HVs and its mechanism of action for the treatment of ischemic stroke. Reprinted with permission from Xu et al. [94], Feng et al. [97], Dong et al. [100]

Xu et al. [94] extracted platelet membrane from whole blood of mice, wrapped it around the polymer core of poly(lactide-co-glycolic) acid (PLGA), and then combined recombinant tissue plasminogen activator (rt-PA) with the sulfhydryl group on the platelet membrane through a bifunctional maleimide connector to form PNP-PA nanoparticles (Fig. 2A). As platelets are involved in the process of thrombus formation under pathological conditions, they can be targeted to damaged vessels, to activate internal signaling pathways and to secrete relevant pro-thrombotic cytokines to accelerate thrombus formation [95].They also demonstrated that PNP-PA nanoparticles can preferentially bind activated platelets, and a variety of receptors on the platelet membrane, such as CD41, CD61, CD62p, etc., are involved in the molecular recognition of PNP-PA and thrombus, thus achieving the targeted delivery of thrombus. Based on such a drug delivery strategy, in addition to paying attention to the properties of cell membranes themselves, we can explore their roles in pathogenesis or repair processes under certain pathophysiological conditions to screen for more suitable delivery bionanomimetic membranes, of which platelet membrane is a typical example.

The focus of stroke is not only involved in vascular destruction and repair, but also an environment in which brain microvessels are in an inflammatory storm. In a short period after the occurrence of stroke, it will lead to the accumulation of aerobic metabolites and the production of free radical oxygen, and peripheral immune cells will be recruited to the brain for the first time [96]. Based on such a phenomenon, Feng et al. [97] reported a neutrophil membrane-encapsulated nanoconjugated enzyme, which was synthesized from ferricyanide and polyethylpyrrolidone using a solid phase reaction method into a nanobased enzyme with enzyme-like activity and a mesoporous structure (MPBzyme), and then coated MPBzyme with differentiated HL-60 cell membrane, resulting in the formation of a nanoconjugated enzyme with neutral cell membrane properties (MPBzyme@NCM) (Fig. 2B). The nanomembrane utilized proteins such as integrin β2, LFA-1, and Mac-1 on the neutral cell membrane to bind to adhesion molecules such as ICAM-1 on the endothelial cells of inflammatory brain microvascular after ischemic brain injury [98, 99] to achieve non-invasive targeted delivery of MPBzyme@NCM and very good therapeutic effects have been achieved.

In addition, since neutrophils play a central role in inflammatory injury in stroke, Dong et al. [100] isolated neutrophil-like cells from human leukemia cells (HL-60) and prepared EVs containing cell membrane proteins by nitrogen cavitation. Then, Resolvin D2 (RvD2) molecules with anti-inflammatory effects were loaded into the EVs(RvD2-HVs) (Fig. 2C). The results showed that the EVs were able to specifically bind to inflammatory cerebrovascular endothelial cells, but not to normal cerebrovascular endothelial cells and reduced cerebral infarct volume, inflammatory factor levels, and neutrophil infiltration, while improving the neurological function of the mice. Therefore, in the application of biomimetic membrane materials in the treatment of stroke, we can return to the pathophysiological mechanism of stroke, explore the trend of some cells or EVs under pathological conditions, so as to select a suitable biomimetic membrane, which is exactly one of the keys to our design of biomimetic membrane materials.

### Alzheimer’s disease (AD)

AD is a neurodegenerative disease that is incurable due to a complex pathogenesis and is the most common form of dementia in the elderly. The pathogenesis includes amyloid beta protein (Aβ) deposition, Tau protein aggregation, neuroinflammation, etc. leading to neuronal death or cognitive impairment. The global population is increasing, accompanied by a serious aging of the population. Currently, the number of people suffering from AD is now over50 million worldwide, and will continue to rise at a rapid rate in the future. Age, gender, poor lifestyle, genetics, etc. are all high risk factors for AD [101]. In order to treat the pathological changes of AD, drugs usually need to be transported directly into the brain tissue and act on the brain cells to achieve the best possible efficacy and minimize the side effects of the drug. However, the delivery of effective drugs to the central CNS requires several critical processes, including prolonging the circulating half-life, crossing the blood-brain barrier (BBB), and uptake by target cells. Currently, there are significant difficulties and challenges with these processes, making it difficult to effectively treat many CNS disorders, including AD.

**Fig. 3:**
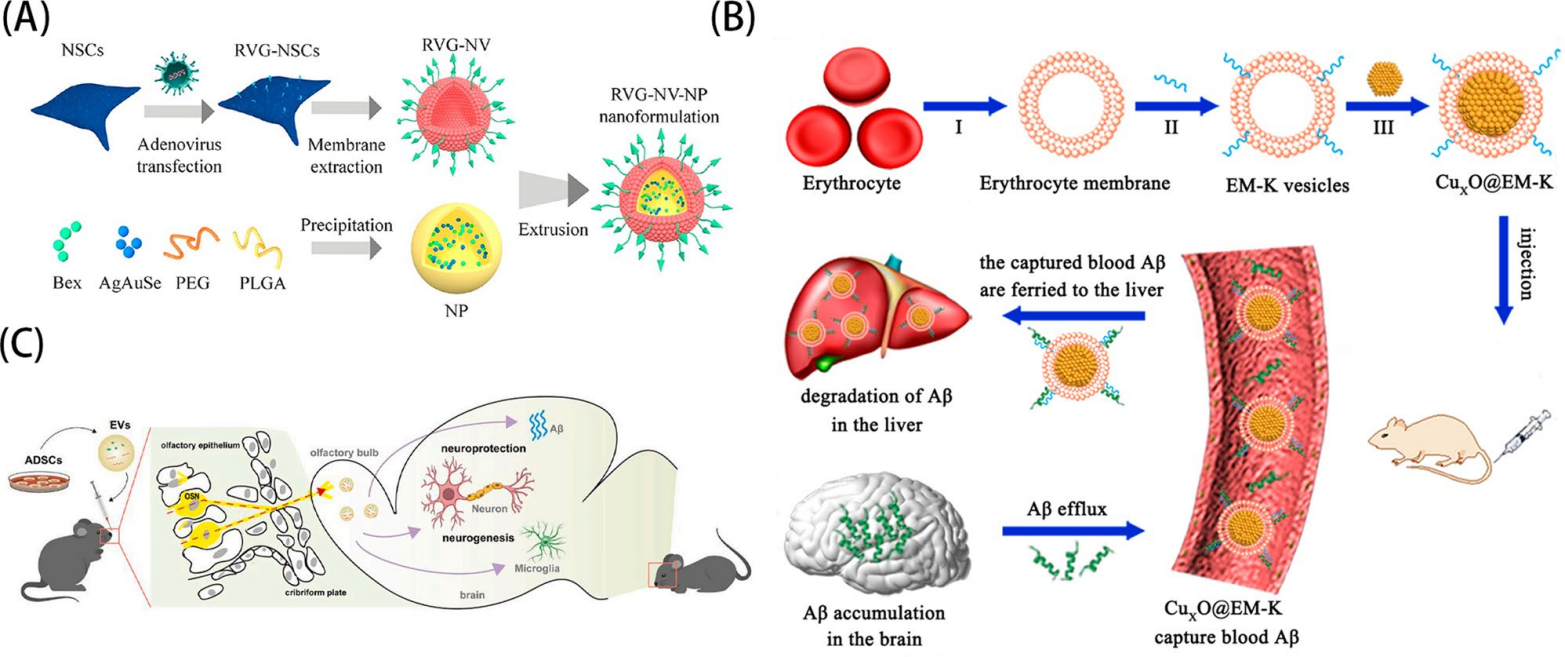
(**A**) The schematic diagram of the preparation process of RVG-NV-NPs. (**B**) The schematic diagram of the synthesis of CuxO@EM-K and its clearance of peripheral Aβ. (**C**) The schematic diagram of the therapeutic mechanism of EVs in APP/PS1 transgenic mice. Reprinted with permission from Huang et al. [45], Ma et al. [103], Ma et al. [104]

Huang et al. [45] reported a study of NPs formed by wrapping a drug (Bex) capable of scavenging soluble Aβ and a type of quantum dots (AgAuSe QDs) capable of luminescence in the near-infrared two region in polymers (PLGA and DSPE-PEG). Through genetic engineering, they made neural stem cells (NSC) express a targeting peptide (RVG) that specifically recognizes cerebrovascular endothelial cells and neuronal cells and fused it to lysosome-associated membrane glycoprotein 2b (Lamp2b) to form a Lamp2b-RVG fusion protein, then, the cellular membranes of Lamp2b-RVG-expressing NSCs were extracted (RVG- NV) and wrapped on the surface of NPs to form RVG-NV-NPs nanopreparations. (Fig. 3A) Through in vitro and in vivo experiments, it was verified that RVG-NV-NPs could reach the brain through the BBB smoothly and effectively reduce Aβ levels to achieve therapeutic effects. In this therapeutic strategy, we got some inspiration that brain-derived cells (such as NSCs) have a natural homing effect (i.e., the ability to converge to the brain), which can help their contents to enter the brain through the blood-brain barrier. So whether other cell membranes in the CNS (astrocytes, microglia, neuronal cells, brain endothelial cells, etc.) can also be used in the treatment of Alzheimer’s disease or other CNS disorders, where their surface membrane proteins, physiological effects, etc., may have adverse effects on the brain, are all things we need to consider. In addition, genetically engineered modifications (i.e., RVG) were performed on NSCs in this study. In recent years, the modification of targeting peptides on biomimetic membranes seems to be a novel and fashionable design, and the targeting peptides (such as T7, D-T7, GSH, TGN, CGN, and TAT) enhance the targeting of biomimetic membrane materials to the BBB, which has great development prospects [102].

Ma et al. [103] designed a biomimetic nanoenzyme called Cu_x_O@EM-K (Fig. 3B), which consists of a Cu_x_O nanoenzyme core and a red blood cell membrane modified with the Aβ-targeting peptide KLVFF. Copper oxide nanozymes have a variety of antioxidant enzyme-like activities, which can alleviate the oxidative damage of erythrocyte membrane induced by Aβ and stabilize the outer erythrocyte membrane. Aβ targeting peptide KLVFF is a pentapeptin derived from Aβ, which can act as a specific ligand of Aβ and play A synergistic role with the erythrocyte membrane to effectively capture Aβ in the blood. In this study, the researchers’ starting point was not to deliver drugs to the brain, but to treat Alzheimer’s disease from the peripheral perspective. According to reported studies, Aβ is also accumulated in the brain and peripheral organs, including the blood, and affects the progression of the disease. Thus, clearance of peripheral Aβ can promote massive efflux of Aβ from the brain into the blood through a sink effect and lead to a rapid reduction in brain Aβ levels. In addition, they also modified the Aβ-targeting peptide KLVFF on the cell membrane to achieve precise targeting. It can be seen that the application of targeted peptides in central nervous system diseases has a strong potential.

Ma et al. [104] used EVs derived from adipose-derived mesenchymal stem cells (ADSCs) (Fig. 3C) because these EVs are enriched in a variety of proteins with neuroprotective and neurogenic activities. Through intranasal administration, EVs can rapidly and efficiently enter the brain, mainly accumulate in neurons, and promote neurogenesis and neuroprotection. Also learning from the therapeutic strategy of stem cell transplantation, EVs were applied to the treatment of Alzheimer’s disease. They chose EVs derived from ADSCs, which were more easily available, and directly acted on the brain by internal nasal administration [105], bypassing the blood-brain barrier. This approach not only provides a novel therapeutic avenue for AD, but also provides a new strategy for restoring cognitive function in AD patients by promoting neurogenesis and neuroprotection.

### The CNS tumor

The CNS tumors are those that occur in the CNS sites such as the brain, spinal cord, cranial nerves, and meninges, which are commonly found in children, adolescents, and young adults [106], with a high prevalence of several major tissue types such as gliomas, medulloblastomas, CNS lymphomas, and meningiomas. CNS tumors are highly lethal and disabling diseases that have a serious impact on the quality of survival and functioning of patients and also place a heavy burden on the health system. Diagnosis and treatment of CNS tumors require highly specialized techniques and equipment that are not commonly available in many regions. In 2016, there were approximately 330,000 new cases of CNS tumors, 227,000 deaths, and 770,000 disease burdens (in terms of DALY) worldwide. The incidence and mortality rates of CNS tumors vary significantly between regions and countries, and are correlated with sociodemographic indices (SDI). In general, morbidity increases with increasing SDI, while mortality decreases with increasing SDI, reflecting differences in diagnostic and therapeutic levels in different regions. The risk factors for CNS tumors are unclear, and only a few factors (such as ionizing radiation, genetic syndromes, and allergic diseases) have been consistently associated with them [107]. Cell membrane-coated nanoparticles (CNPs) can be used for drug delivery, phototherapy, and immunotherapy of tumors, and a variety of CNPs have been shown to have anti-tumor effects in animal models. CNPs can utilize specific receptors or ligands of the cell membrane to achieve targeted delivery of tumors, or can use immune regulatory factors of the cell membrane to activate or inhibit the function of immune cells, or directly interact with immune cells to interact and promote the presentation of tumor antigens and the expansion of T cells [108].

**Fig. 4:**
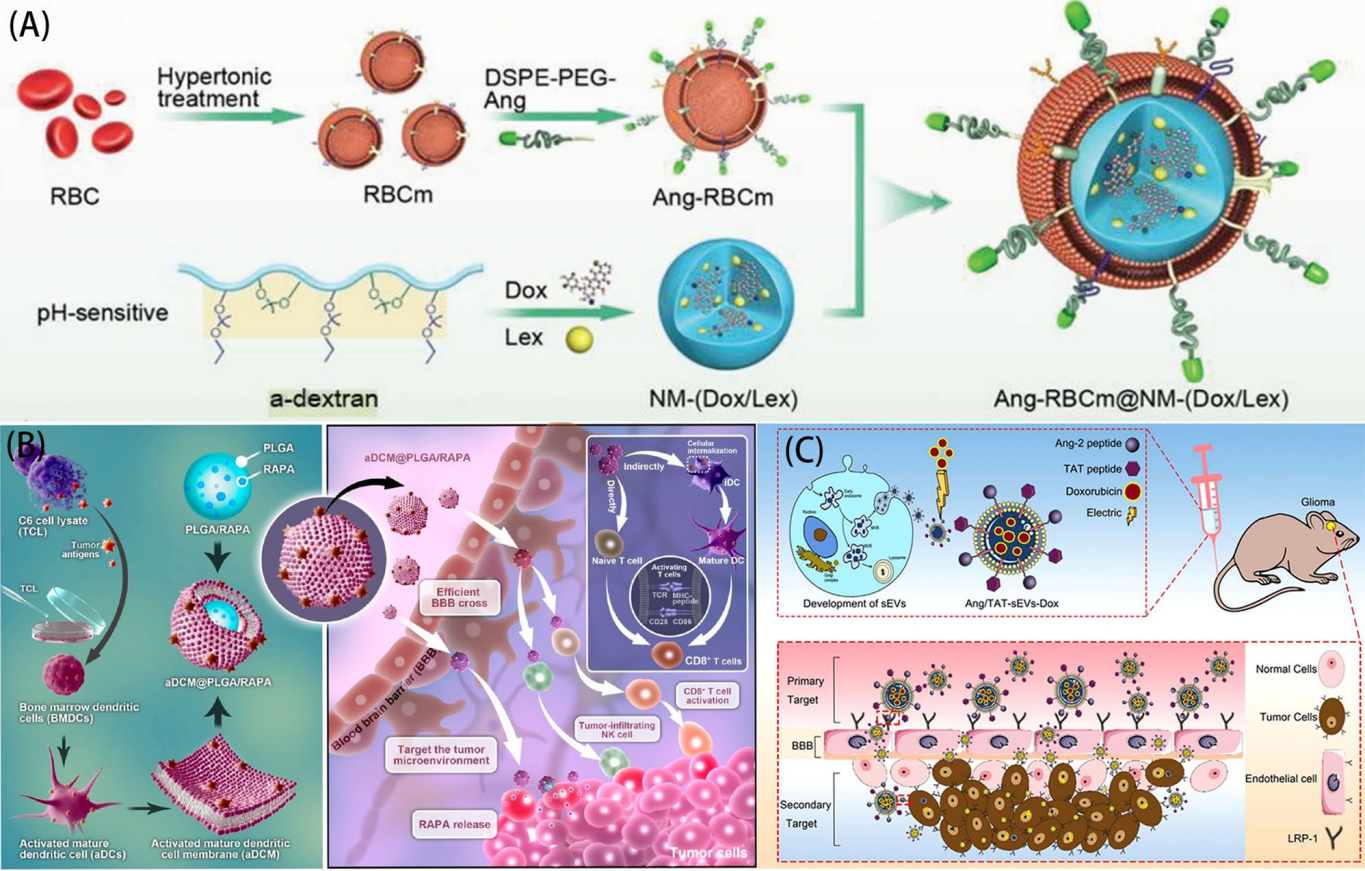
(**A**) The Schematic diagram of the preparation process of Ang-RBCm@NM-(Dox/Lex). (**B**)The schematic illustration of the preparation process of aDCM@PLGA/RAPA and its mechanism of action in the treatment of glioma. (**C**) The schematic diagram of the preparation process of Ang/TAT-sEVs and the mechanism by which it acts on gliomas in vivo. Reprinted with permission from Zou et al. [109], Ma et al. [46], Zhu et al. [113]

Zou et al. [109] reported a multifunctional biomimetic nanomedicine in which a red blood cell membrane (RBCm) was fused with a pH-sensitive nanomedicine nucleus (NM) containing the anticancer drug Doxorubicin (Dox) and the blood-brain barrier modulator Lexiscan (Lex) by mechanical extrusion to form RBCm@NM-(Dox/Lex). (Fig. 4A) At the same time, the NPs were loaded with ph-sensitive chemotherapeutic drugs Dox and Lex, which achieved the triggered release in the tumor microenvironment, inhibited the growth of tumor cells, and prolonged the life span of model mice. In this study, erythrocyte membrane was selected as the carrier, because RBC membrane has high biocompatibility and low immunity, which can prolong the life of NPs in the blood, and the targeting peptide Angiopep-2 (Ang) is modified on the erythrocyte membrane to enhance the targeting ability of the drug to the BBB. In this research strategy, the use of pH-responsive chemotherapeutic drug Dox and blood-brain barrier modulator Lex is a highlight to learn from. Lex can transiently open the BBB and enhance the permeability of NPs to the brain [110], so Lex can be added to the design of biomimetic membrane materials to improve the efficiency of NPs through the BBB. In addition, drug responsiveness is also very important, for example, PH response improves the precision of drug delivery for release at acidic sites of injury (e.g., SCI, TBI) [111]. These are important factors for us to design biomimetic membrane materials.

Ma et al. [46] extracted dendritic cells (DCs) from mouse bone marrow and induced their differentiation with GM-CSF and IL-4. The DCs were co-cultured with glioma cell lysate to mature DCs and express tumor antigens. Dendritic cell membranes (aDCM) were wrapped around drug-loaded(Rapamycin RAPA) poly(lactic-co-glycolic acid) (PLGA) NPs to form aDCM@PLGA/RAPA nanoplatforms (Fig. 4B). The aDCM@PLGA/RAPA nanoplatforms have two features, that is, the tumor antigens on the aDCM can be isotype-recognized by the homologous tumor cells, which enhances the tumor-targeting ability of the NPs; and the presence of immune cell membrane proteins on the aDCM facilitates this nanoplatform to cross the blood-brain barrier (BBB) and target the tumor tissues. In this research strategy, they connected tumor immunotherapy with the application of biomimetic membranes. The activated aDCM not only provides a biomimetic membrane, but also stimulates the immune response of the body, promotes the activation and proliferation of T cells and NK cells, and inhibits the exhaustion of T cells, thus killing tumor cells directly or indirectly [112]. aDCM and RAPA realize the synergistic effect of two treatment modes, namely immunotherapy and chemotherapy, which provides a new idea for the treatment of central tumors with biomimetic membrane materials.

Zhu et al. [113] demonstrated that small HEK293T derived extracellular vesicles (Ang/ TAT-sevs) were doubly functionalized with a targeting peptide Ang and a cell-penetrating peptide (TAT), Ang is an active target peptide with high affinity for low-density lipoprotein receptor-related protein-1 (LRP1) (LRP1 is highly expressed in glioma cells (U87MG)) and has high brain penetration [114]. TAT peptide is a highly efficient cell-penetrating peptide that can penetrate the plasma membrane and nuclear envelope of most living cells [115], and Dox is loaded into sEVs by electroporation (Ang/ TAT-SEVs-DOX) for efficient and specific glioma therapy. The innovation of this study is to exploit the natural properties and cell penetration ability of sEVs to achieve efficient targeting of BBB and glioma by dual modification with Angiopep-2 and TAT peptides. This approach significantly increases the efficiency of drug delivery and therapeutic efficacy while reducing side effects.

## Conclusion and outlook

In summary, the various advantages of bionanomaterials make them one of the potential delivery modes for the treatment of CNS diseases. Biomimetic membrane materials can give NPs for drug delivery the privilege of “not being eaten” in vivo and can easily pass through the BBB. Different biomimetic membranes can be endowed with different biological functions. Therefore, there are many factors that affect the selection of biomimetic membrane, such as cell subtype, cell pathophysiology, stem cell transplantation for reference, natural homing of cells, tumor cell homology recognition, etc., and even the pathogenesis of disease can be the key to choose which type of biomimetic membrane.

In recent years, engineered and functionalized biomimetic membrane materials have emerged as an emerging platform for drug delivery. Genetic engineering or chemical modification of biomimetic membranes, that is, modifying the surface of biomimetic membranes with targeted peptides or exogenous substances to stimulate cells to make changes in their cell membrane function, enhances the targeting, stealth, and drug-carrying capacity of biomimetic membrane materials and can be precisely delivered to the site of injury of CNS disorders, which provides new and effective therapeutic strategy options.

To date, the technology of biomimetic membrane materials for drug delivery is still in the early stages of novelty, with the most effective evidence only in cellular or animal models. The preparation technology of biomimetic membrane materials is still not mature enough, and the preparation process may be more complicated, which requires fine technology and condition control, thus making it difficult to produce on a large scale. In terms of clinical applications, there are no examples of biomimetic membrane materials applied to the clinic, and the effectiveness of biomimetic membrane materials in large animals (such as non-human primates) has not been further evaluated. Potential safety and biocompatibility issues in the clinical setting cannot be ruled out and require rigorous biosafety assessment.

Although biomimetic membrane materials still have certain limitations in clinical applications, their therapeutic effects in cellular or animal models of CNS disorders are obvious and are significantly more efficient than the therapeutic effects of traditional drug delivery methods. It is believed that with the gradual maturation of this technology, biomimetic membrane materials will continue to provide new possibilities in the diagnosis and treatment of CNS diseases, which is worth looking forward to.

## Data Availability

The data used to support this review are included within the article.
